# Synthesis, local structure and optical property studies of α-SnS microrods by synchrotron X-ray pair distribution function and micro-Raman shift

**DOI:** 10.1039/d0ra03586f

**Published:** 2020-06-03

**Authors:** U. P. Gawai, D. K. Gaikwad, S. L. Patil, K. K. Pandey, N. P. Lalla, B. N. Dole

**Affiliations:** Department of Physics, YCSPM's, DDSP, Arts Commerce and Science College Erandol Jalgaon-425109 India upgawai.phys@gmail.com; Department of Physics, ACS College Dharangaon-425105 India; High Pressure & Synchrotron Radiation Physics Division, Bhabha Atomic Research Centre Mumbai M.S. India; UGC-DAE CSR University Campus, Khandwa Road Indore-452017 India; Advanced Materials Research Laboratory, Department of Physics, Dr Babasaheb Ambedkar Marathwada University Auranagabad-431004 M.S. India

## Abstract

A hydrothermal synthesis method was employed for the preparation of tin sulfide (α-SnS) microrod samples (SnS-A and SnS-B) using ethylenediamine and deionized water as the surfactant at ratios from 50 : 50 to 100 : 00. The atomic structures of the α-SnS microrods were studied using atomic pair distribution function (PDF) analysis and total synchrotron X-ray scattering data. The synchrotron X-ray diffraction (ScXRD) patterns and PDF data reveal that the structure of the SnS microrods is orthorhombic. From the refinement of the PDF, the first and second peaks correspond to nearest (Sn^2+^–S^2−^) and second nearest distances (Sn^2+^–Sn^2+^) of 2.546 (0.003) Å and 4.106 (0.004) Å, and 2.527 (0.005) Å and 4.087 (0.006) Å for SnS-A and SnS-B samples, respectively. The TEM results show that samples SnS-A and SnS-B have a microrod structure, with microrod diameters of 800 nm and 500 nm with lengths of tens of micrometers, respectively. The SnS-A and SnS-B samples show a direct band gap of 1.6 eV and 2 eV, respectively, using the Kubelka–Munk transformation of the UV-visible spectra. The micro-Raman spectra of the SnS-A and SnS-B microrods exhibited an Ag mode of SnS at 228.4 and 223 cm^−1^, respectively. The second peaks at 306.7, and 309 cm^−1^ are associated with the secondary phases of the SnS_2_ phase, whereas the third broad peaks at 616.5, and 613 cm^−1^ revealed that there was a deformation mode of sulfate in the SnS-A and SnS-B samples.

## Abstract 2

The PDF refinement shows layer structure of SnS-A with two distinct bond lengths, one nearly parallel to the ‘*a*’ axis and another perpendicular to the ‘*a*’ axis, it corresponds to bond lengths of 2.62528 (38) Å and 2.66204 (03) Å.

## Graphical abstract

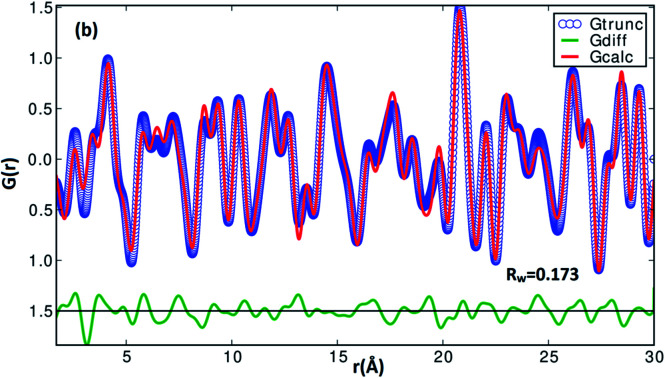

## Introduction

Tin sulfide (SnS) has attracted much attention in recent years due to its narrow bandgap that displays optical activity in the near-infrared (NIR) region, with potential applications in photovoltaic devices and NIR detectors.^1,2^ Tin sulfide is one of the most abundant, cheapest, eco-friendly and, due to its direct band gap, is used for applications in solar cells,^3^ lithium storage,^4^ hydrogen storage,^5^ thermoelectric and photonic devices^6^ and so on. Tin sulfide has two structures one is π-SnS cubic and the other is an α-SnS orthorhombic structure. Orthorhombic SnS crystallizes in space group *Pnma* in the GeS (B16) type structure with lattice parameters of *a* = 11.200(2) Å, *b* = 3.987(1) Å and *c* = 4.334(1) Å.^6^ The Sn and S occupy the Wyckoff position 4c (*x*, 1/4, *z*) with fractional coordinates of *x* = 0.1194(1), *z* = 0.1198(2) for tin and *x* = 0.8508(3), *z* = 0.4793(8) for sulfur.^6^

A large amount of research is carried out on the synthesis and characterization of 1D well-designed SnS materials.^4,7^ The 1D structure can be fabricated by number of techniques, such as surfactant-assisted techniques,^8^ hydrothermal methods,^9^ noncovalent self-assembly,^10^ chemical vapor deposition,^11^ thermal decomposition,^12^ and so on. However, further development of these techniques for practical routes to make large quantities of materials with a porous 1D structure with accurate size, shape control, rapidly and reasonably low costs, are still a great challenge.

The determination of the crystalline structure of a solid is a key part of materials' science, and for this, the powder diffraction method is widely used. It is an excellent method but challenges exist when determining the local structures of complex materials. Nowadays, synchrotron X-ray diffraction (ScXRD) with fast computing methods has been used for the determination of the atomic-scale structure of materials. The ScXRD with a pair distribution function (PDF) is one of the most powerful tools for determining the local structure of atoms with shorter and moderate lengths.^13,14^ The PDF is one of the most versatile methods which can be applied to any materials.^15–17^ This method has numerous applications for the analysis of the structures of materials for determining crystal phase and unit-cell parameters and for quantifying various types of disorder or defects.^18–20^ The PDF gives a histogram of interatomic separations in a crystal/material and represents a weighted bond length distribution. The Bragg peaks arise due to the translational symmetry present in the samples.

The total scattering technique, which outlines the Bragg peaks with their position and intensity, reveals the structure, and diffuse scattering with deviation gives a perfect lattice. The PDF *G*(*r*) is a 1D function that oscillates around zero and indicates, *via* the peak positions, the distances of separation of pairs of atoms. The peak positions of the PDF correspond to the distribution of the distances in the material. A negative valley in the PDF corresponds to the real-space vector not having atoms at either end, and for this reason, the PDF resembles a Patterson function and is therefore widely applicable for X-ray crystallography studies. Fourier analysis of the total scattering is known as atomic PDF analysis, meaning that the PDF is the Fourier transform of the scattering intensity.^19,20^ Recently, rapid developments in science and technology have posed new challenges for the study of atomic-scale structures using ScXRD. Because of the crystallinity of certain materials, it does not always act as a perfect grating, therefore the ScXRD patterns can show both Bragg peaks and diffuse components.^17–21^ The PDF uses both the Bragg peaks and diffuse components to reveal the total scattering in the ScXRD data. The PDF is obtained from a total scattering powder diffraction pattern *via* a Fourier transform analysis, because the total scattering pattern is composed of Bragg peaks as well as diffuse scattering contributions. The PDF peak position contributes to the average distance separation of the pair, and its integrated intensity reveals the number of coordinate atoms, and its width and shape indicate the static or dynamic disorder in the pair.^22–24^ Also Raman spectroscopy is a vibrational technique routinely used to provide local structural information at the atomic scale. Herein, the hydrothermal synthesis of α-SnS microrods using ethylenediamine the surfactant is reported. Also, different studies were carried out on the α-SnS microrods, such as ScXDR, PDF, transmission electron microscopy (TEM), micro-Raman and UV-Vis spectroscopy.

## Experimental details

A hydrothermal synthesis method was employed for the synthesis of the SnS-A and SnS-B microrods. In a typical synthesis process, a 100 ml Teflon-lined stainless steel autoclave was used. All of the chemicals were analytical grade and used without any further purification. The appropriate amount of tin chloride dihydrate (SnCl_2_·2H_2_O, 0.1 M), and thiourea (0.2 M) was dissolved separately in a 50 : 50 and 0 : 100 ratio of deionized (DI) water and ethylenediamine solution as surfactant. Then, these two mixtures were stirred for 20 min at room temperature and these white colored solutions were transferred separately to an autoclave. The autoclaves were sealed and kept on a hot plate at a temperature of 185 °C for 12 h and then allowed to cool naturally. During the formation of the SnS phase, the initial white color changes to a dark brown color. The product obtained was purified, washed several times with DI water, and then filtered. The final product was dried in air at 60 °C for 2 h.

### Characterization of samples

The ScXRD patterns were recorded using angle dispersive X-ray diffraction (ADXRD) with a wavelength of 0.4414 Å at an energy of ∼28 keV from the beamline (BL-11) at INDUS-2, Raja Ramanna Center for Advanced Technology (RRCAT), Indore, India. The detailed experimental procedure for obtaining ScXRD data was reported previously.^13–15^ Samples of SnS-A and SnS-B were sealed in Kapton foil for the ScXRD measurements. The sample was mounted orthogonally in the path of the beam with a distance between the samples and detector of 211 mm. The IP was exposed for each sample at 100 s and then a long 5400 s to obtain the total scattering data signals. These 2D images were calibrated using a standard CeO_2_ sample. The scattering signal of the samples was measured independently and subtracted from the background (Kapton foils, air) in the data. The TEM measurements and selected-area electron diffraction (SAED) patterns were obtained at an accelerating voltage of 200 kV. The energy dispersive spectra (EDS) were collected using a Jeol JSM-6360 SEM. The UV-Vis reflectance spectra were acquired using a PerkinElmer Lambda 950 UV-Vis spectrophotometer. The Raman spectra were recorded using a Jobin Yvon Horiba LABRAM-HR micro-Raman system in the visible region.

## Results and discussion

### Crystallographic study

**Fig. 1:**
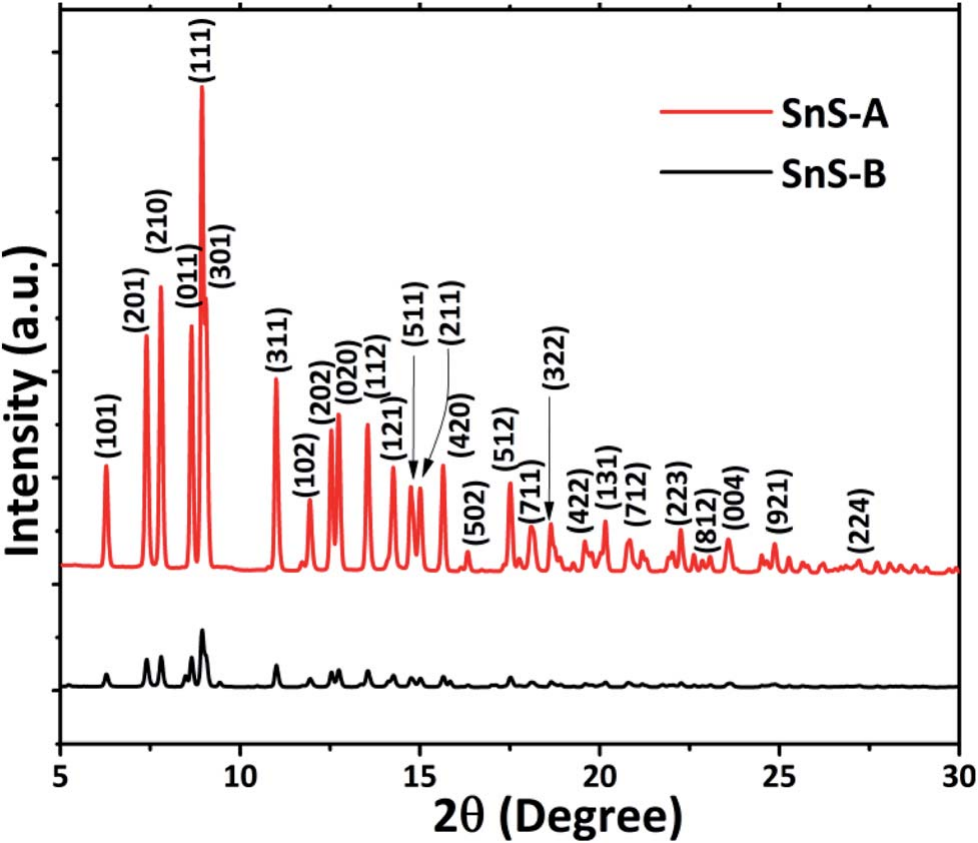
Synchrotron X-ray diffraction patterns of α-SnS samples: SnS-A and SnS-B.

**Fig. 2:**
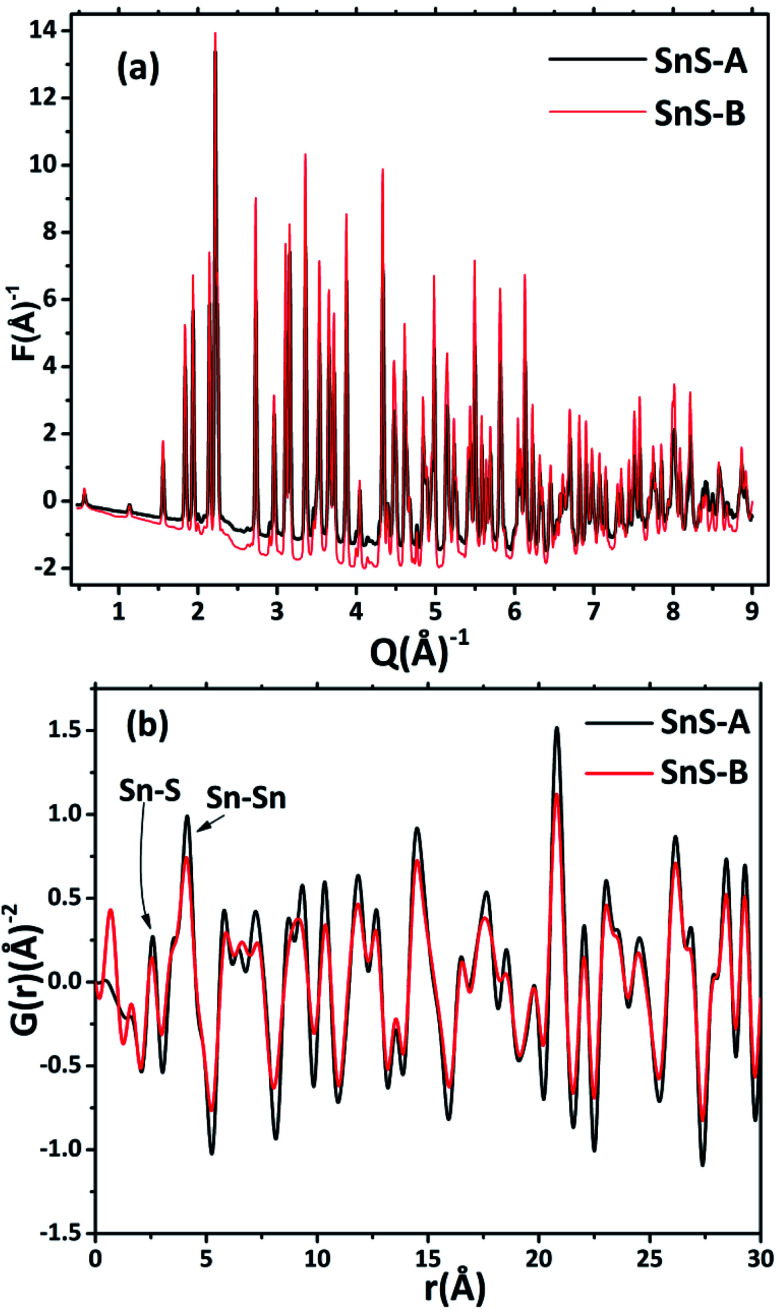
(a) The experimental reduced structure functions *F*(*Q*) of α-SnS microrods, and (b) the corresponding pair distribution functions obtained by Fourier transformation of the sample data.

**Fig. 3:**
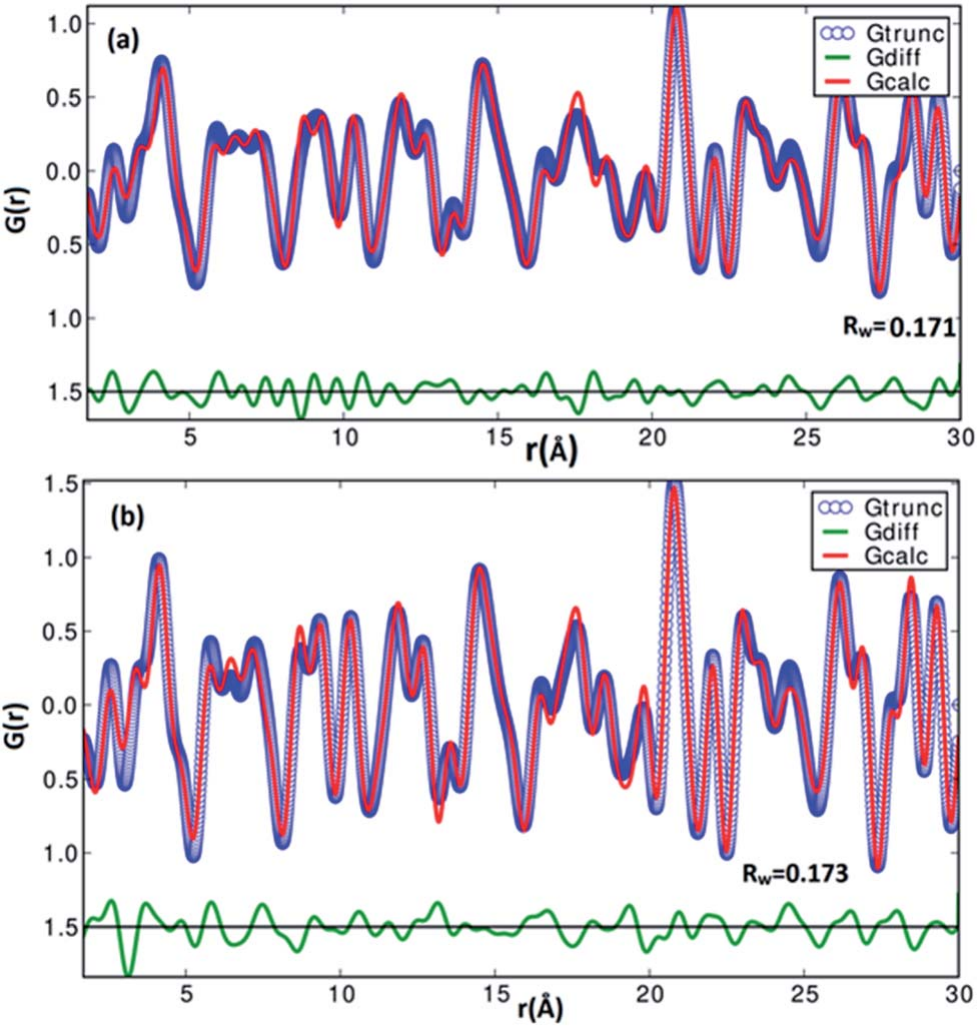
The experimetal and refined PDFs *G*(*r*) of α-SnS samples (a) SnS-A and (b) SnS-B, which correspond to orthorhombic structures.

The ScXRD patterns of the α-SnS samples are shown in Fig. 1. The ScXRD data were used to obtain the PDF from the AD/ED-XRD BL-11 beamline at RRCAT. The 2D images were recorded on a marXperts mar345 2D image plate camera with an imaging plate of 50 × 30 mm, using a compound refractive lens.^25^ The scattering signal obtained from the sample was measured independently and subtracted from the background signal during data reduction. Then the collected 2D diffraction images were combined and subjected to geometric correction, integrated and converted to intensity *versus* 2*θ* using the FIT2D program.^26^ To investigate the local structure of the SnS microrods, the ScXRD and PDF techniques were combined. For the total scattering structure function *S*(*Q*) and PDF, the data were corrected and normalized using the program PDFgetX3-1.1 (ref. 27) and the results are shown in Fig. 2(a) and (b).^17^ In the Fourier transform step, to get from *S*(*Q*) to PDF *G*(*r*), the data were truncated at a finite maximum value of the momentum transfer, *Q*_max_ = 9.5 Å^−1^, and it was found to be optimal.^17^ The SnS material revealed a long range atomic order which was expected from the ScXRD and PDF data because the patterns of the SnS microrods showed well defined Bragg peaks at *Q* = 9.5 Å^−1^. The ScXRD results revealed high resolution diffraction for structural refinement. From Fig. 1 the diffraction peak of the SnS-A and SnS-B samples could be readily indexed as an orthorhombic structure. There were no secondary phases observed in the samples. The ScXRD results were very close to the standard value card no. JCPDS-89-0253 space group *Pnma* (62), with lattice constants for SnS-A of *a* = 11.312 Å, *b* = 3.971 Å, *c* = 4.305 Å and for SnS-B of *a* = 11.256 Å, *b* = 3.948 Å, *c* = 4.272 Å.^38^ From Fig. 1 the full width at half maxima (FWHM) of the (111) peak (this peak was strongest and narrowest of all the peaks), indicated that microrods had formed in the (111) plane. Sample SnS-A seemed to have more crystallinity when compared to SnS-B. This could be inferred from relatively broader peaks in the XRD as well as in the PDF data presented in Fig. 3. Also the TEM results showed that SnS-A seemed to have relatively longer crystals when compared to SnS-B.

A pair distribution function *G*(*r*) was plotted, which revealed the probability of finding a certain distance ‘*r*’ between the atoms shown in Fig. 2(b). It can be seen in Fig. 2(b) and 3 that the as-synthesized samples showed a series of well-defined peaks up to quite a long real-space distance, which meant that the samples exhibited along-range periodic atomic order. The meaning of the pair distribution function *G*(*r*) is the probability of finding the nearest neighbour at a certain distance ‘*r*’ from atoms. In Fig. 2(b) the scan shows sharp and well resolved peaks which suggested high symmetry and well-defined local structures for the SnS samples, which gave the interatomic distances from the peak values. Quantitative structural information was extracted from the refinement of the PDF by comparing data from the observed PDF and the calculated PDF from the models. The PDFgui computer program^27^ was used to fit and simulate the structural model of the experimental PDF, as shown in Fig. 3. From the refinement of PDF, the first and second peaks corresponded to the nearest (Sn^2+^–S^2−^) and second nearest distances of (Sn^2+^–Sn^2+^) at 2.546 (0.003) Å, 4.106 (0.004) Å and 2.527 (0.005) Å, 4.087 (0.006) Å for the SnS-A and SnS-B samples, respectively, with *R*_w_ = 0.17. Here the residual function (*R*_w_) was used to quantify the agreement of the calculated PDF from refinement to experimental data. Results for the modelling of the structures of the samples, concerning the unit cell parameters, atomic displace parameters (ADPs), scale factors, and resolution damping factors (*Q*_damp_) are given in Table 1 but they are slightly different. Also from the refinement from Fig. 3, the α-SnS layer structure has two distinct bond lengths, one nearly parallel with the ‘*a*’ axis (Sn1–S1 or Sn2–S2) and another perpendicular to the ‘*a*’ axis (Sn2–S1). The corresponding bond lengths at 2.62528 (0.0038) Å and 2.66204 (0.003) Å for SnS-A, and 2.62571 (0.005) Å and 2.65981 (0.005) Å for SnS-B matched well with those reported by Liu *et al.*^38^ at (2.687 Å and 2.698 Å) because the α-SnS is a layer structure which has two distinct bond lengths.

**Table 1:** The refined structural parameters of samples SnS-A and SnS-B obtained by PDF analysis

Sample	Structural parameter	Obtained value
SnS-A	*a* (Å)	11.1931 (0.016)
	*b* (Å)	3.98608 (0.0062)
	*c* (Å)	4.32257 (0.0051)
	SnS *U*_11_ = *U*_22_ = *U*_33_	0.0195761 (0.0022)
	*Q*_damp_	0.0199048 (0.0078)
	Scale factor	0.46212 (0.034)
	Sn–Sn	2.62528 (0.0038)
	Sn–S	3.28478 (0.0043)
	*R*_w_	0.171
SnS-B	*a* (Å)	11.1951 (0.024)
	*b* (Å)	3.98104 (0.0085)
	*c* (Å)	4.32122 (0.0073)
	SnS *U*_11_ = *U*_22_ = *U*_33_	0.0262051 (0.0034)
	*Q*_damp_	0.017481 (0.012)
	Scale factor	0.389119 (0.037)
	Sn–Sn	2.62571 (0.0055)
	Sn–S	3.28262 (0.0060)
	*R*_w_	0.173

The growth of a microrod like structure in both samples was observed but SnS-A showed high crystallinity due to the presence of water. In this reaction the water oxidized tin source (Sn^2+^) and ethylenediamine form a tin complex [Sn-(ethylenediamine)_*n*_]^2+^ but due to high pressure and temperature the stability of the ethylenediamine-complex decreased and S^2−^ immediately reacted with it to form the SnS microrods. It is well known that Sn and S combine with the double layered crystal structure which is perpendicular to the *c*-axis. However, in the case of SnS-B, 100% of the ethylenediamine tin source was not immediately oxidized and the correct reaction may not have occurred, and thus, less crystallinity was observed.^39^

### Raman spectroscopy

Micro-Raman analysis was carried out on α-SnS microrods having an orthorhombic structure with eight atoms per unit cell. For the orthorhombic structure, the 24 vibrational modes are represented by the following irreducible representations at the center of Brillouin zone as:

1 *Γ* = 4A_g_ + 2B_1g_ + 4B_2g_ + 2B_3g_ + 2A_u_ + 4B_1u_ + 2B_2u_ + 4B_3u_

**Fig. 4:**
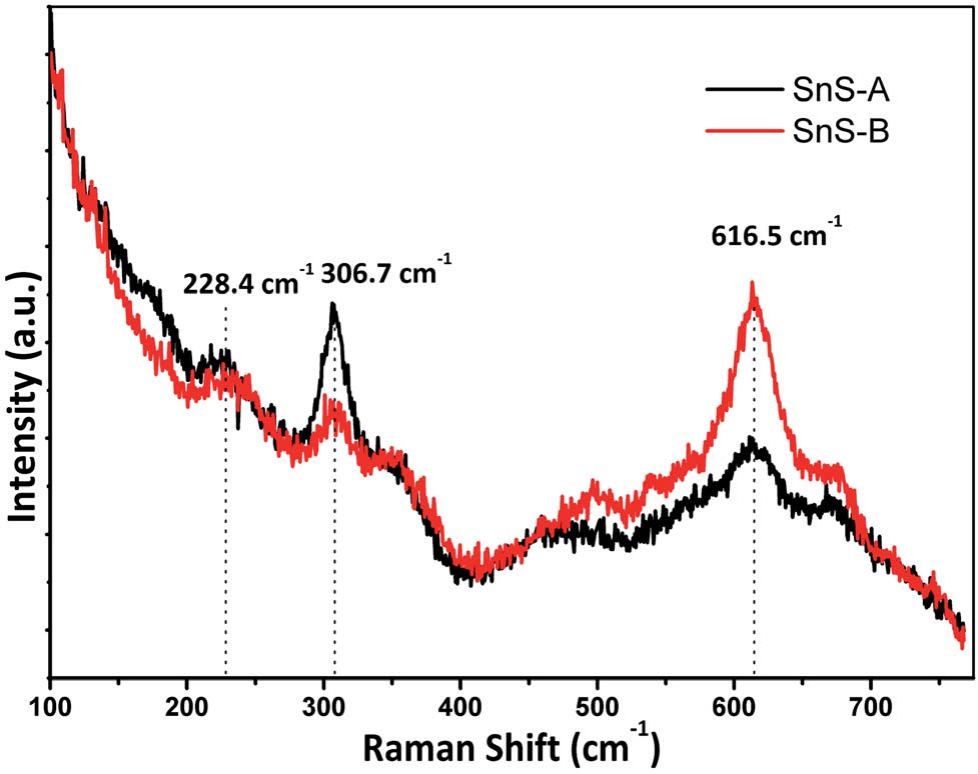
The Raman spectra of samples, SnS-A and SnS-B.

The α-SnS had 21 optical phonons of which 12 were Raman active modes (4A_g_, 2B_1g_, 4B_2g_ and 2B_3g_), seven were infrared active modes (3B_1u_, 1B_2u_ and 3B_3u_) and two were inactive (2A_u_).^28,29^ The micro-Raman spectra for SnS-A and SnS-B microrods exhibiting the Ag mode of SnS at 228.4 and 223 cm^−1^, respectively, are shown in Fig. 4. The reported result was in good agreement with those of Liu *et al.*^30^ which were observed at A_g_ modes at 223, 273.7 cm^−1^ and those of Gou *et al.*^31^ which were observed at A_g_ modes at 189 and 220 cm^−1^ for SnS particles. The next peaks at 306.7 and 309 cm^−1^ associated A_1g_ with the secondary phases of the SnS_2_ phase,^32^ whereas the third set of broad peaks at 616.5, and 613 cm^−1^ revealed the deformation mode of sulfate in SnS-A and SnS-B samples, which was only observed in the bulk materials at a lower temperature, and may be attributed to second-order effects.^32^

### Diffuse reflectance spectroscopic study

**Fig. 5:**
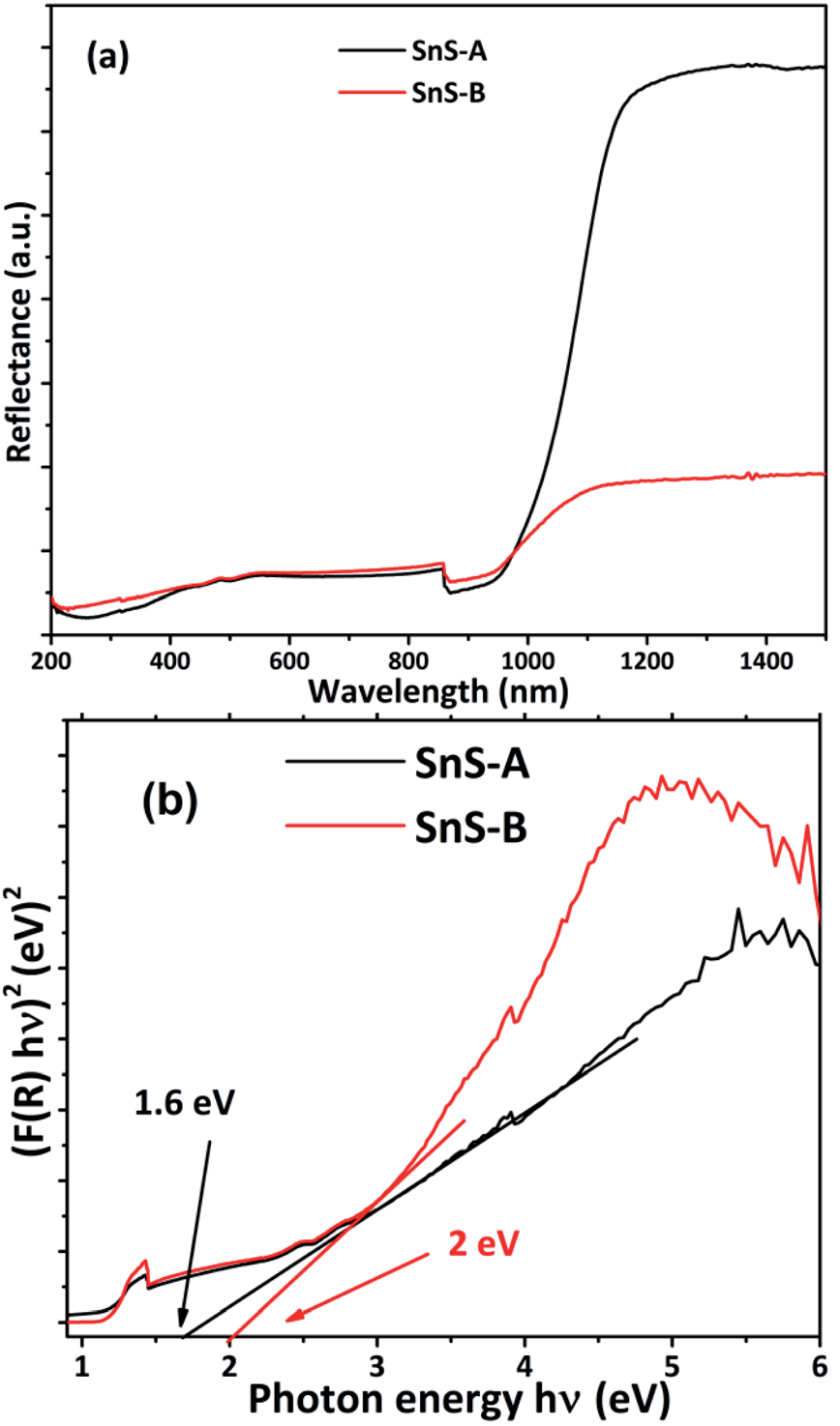
(a) Reflectance spectra of α-SnS samples and (b) Tauc plots of the α-SnS microrod samples.

The energy band gap of a semiconducting material has a significant impact on its optical, electronic and catalytic properties. To investigate the optical properties or bond gap of the samples, UV-diffuse reflectance spectroscopy was carried out and the results are shown in Fig. 5. The UV-diffuse reflectance data of SnS-A and SnS-B samples were processed using the Kubelka–Munk (K–M) equation,^2,5,33^ and were used to further calculate the band gap by using the Tauc equation as follows:^34,35^

2 *F*(*R*) = (1 − *R*)^2^/2*R* = *α*/*s*

where *F*(*R*) is the K–M function, ‘*R*’ is reflectance, ‘*α*’ is the absorption coefficient, and ‘*s*’ is the scattering coefficient.

3 (∝*hν*)^1/*n*^ = *A*(*hν* − *E*_g_)

**Fig. 6:**
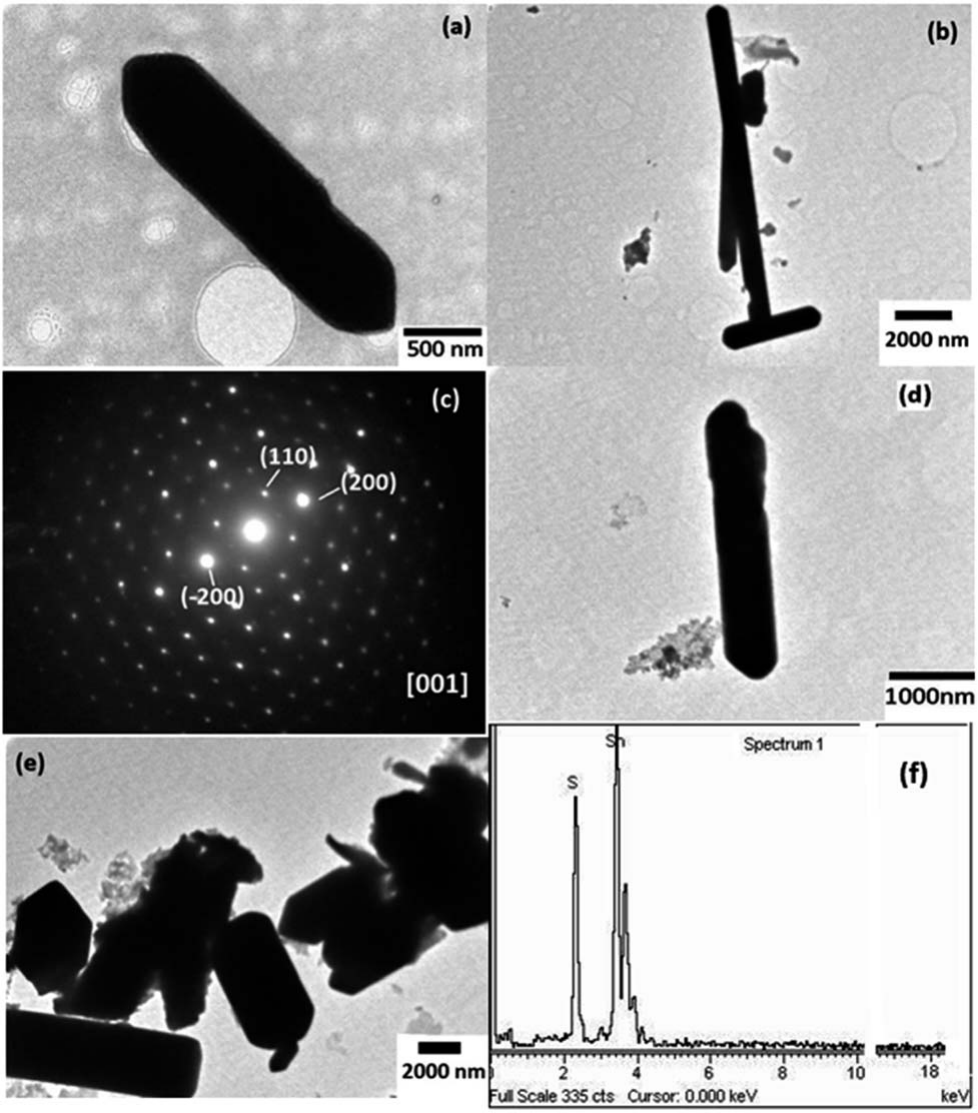
(a and b) TEM images of SnS-A and (c) the SAED pattern taken along the [001] zone of a single microrod of SnS-A. The (110) and (200) spots are indicated. (d), (e) TEM images of SnS-B samples. (f) shows the EDS spectra corresponding to the SnS-A sample.

Where *h* is Planck's constant, *ν* is the vibration frequency, and *E*_g_ is bandgap and the value of *n* = 1/2 for direct band semiconductors and *n* = 2 for indirect band semiconductors. The absorbance data were fitted for *n* = 1/2 as shown in Fig. 5(b). The band gap (*E*_g_) was calculated by extrapolation of the linear region of the Tauc plot, which showed that the band gaps of SnS-A and SnS-B samples, were 1.6, and 2 eV respectively. But bulk SnS had a bandgap value of 1.3 eV which was significantly less than the value obtained in the experimental results, and this was observed to be due to the confinement effect of the rods in the *c* direction.^36^ The experimental results were in good agreement with those of Cho and Sung (1.6 eV),^7^ and Das and Datta (1.81 eV).^37^ The SnS-A and SnS-B samples showed a blue shift, due to a decrease in the average diameter and length of the rods during growth, as shown in Fig. 6.

### Morphological study

The TEM images of the as-synthesized samples are shown in Fig. 6. It showed the formation of non-uniform micro sized rods. The average diameter of the samples, SnS-A and SnS-B was found to be 800 and 500 nm, respectively, with lengths of tens of micrometers. Fig. 6(c) shows the SAED spot patterns of the SnS-A sample. The SAED spot pattern suggested the formation of single crystal SnS-A microrods and the spots were indexed at the (110) and (200) planes along the [001] zone. Fig. 6(f) shows the compositional analysis of the as synthesized samples using EDS analysis. The emission peaks of tin (Sn) and sulfur (S) could be clearly seen. It was confirmed that only Sn and S elements were present in the sample. A EDS spectrum revealed that the atomic wt% of S was 43.43 and for Sn was 56.57.

## Conclusions

Tin sulfide microrods were successfully synthesized by a hydrothermal method using ethylenediamine and deionized water as the surfactant. The atomic structures of the as-synthesized samples were studied using atomic pair distribution function analysis and total synchrotron X-ray scattering data. From the ScXRD patterns and PDF analysis, it is confirmed that the samples have an orthorhombic structure. The TEM micrographs clearly reveal the rod-like structures of the samples. The direct energy band gap of samples was evaluated using UV-Vis spectra. The micro-Raman spectra of SnS-A and SnS-B microrods exhibited an A_g_ mode of SnS at 228.4 and 223 cm^−1^, respectively, whereas the third broad peaks at 616.5, and 613 cm^−1^ revealed the deformation mode of sulfate in the SnS-A and SnS-B samples. From the refinement of the PDF, the α-SnS layer structure has two distinct bond lengths, one nearly parallel with the ‘*a*’ axis (Sn1–S1 or Sn2–S2) and another perpendicular to the ‘*a*’ axis (Sn2–S1), corresponding to bond lengths at 2.62528 (0.0038) Å and 2.66204 (0.003) Å for SnS-A, and 2.62571 (0.005) Å and 2.65981 (0.005) Å for the SnS-B sample. Comparing the SnS-A and SnS-B samples, the water and ethylenediamine (50 : 50) surfactant solution (SnS-A) was preferred from a technological point of view, because it improved the crystallinity.

## Conflicts of interest

There are no conflicts to declare.

## Supplementary Material
